# The Key Role of GSH in Keeping the Redox Balance in Mammalian Cells: Mechanisms and Significance of GSH in Detoxification via Formation of Conjugates

**DOI:** 10.3390/antiox12111953

**Published:** 2023-11-01

**Authors:** Sofia K. Georgiou-Siafis, Asterios S. Tsiftsoglou

**Affiliations:** Laboratory of Pharmacology, Department of Pharmaceutical Sciences, School of Health Sciences, Aristotle University of Thessaloniki (AUTh), 54124 Thessaloniki, Greece; sgeorgi@pharm.auth.gr

**Keywords:** GSH redox cycle, GSH S-conjugates, GSH adducts, GSH metabolism, xenobiotics, metabolic signatures, thioether bonds, GSH-hematin adducts, hemolytic disorders, GSTs

## Abstract

Glutathione (GSH) is a ubiquitous tripeptide that is biosynthesized in situ at high concentrations (1–5 mM) and involved in the regulation of cellular homeostasis via multiple mechanisms. The main known action of GSH is its antioxidant capacity, which aids in maintaining the redox cycle of cells. To this end, GSH peroxidases contribute to the scavenging of various forms of ROS and RNS. A generally underestimated mechanism of action of GSH is its direct nucleophilic interaction with electrophilic compounds yielding thioether GSH S-conjugates. Many compounds, including xenobiotics (such as NAPQI, simvastatin, cisplatin, and barbital) and intrinsic compounds (such as menadione, leukotrienes, prostaglandins, and dopamine), form covalent adducts with GSH leading mainly to their detoxification. In the present article, we wish to present the key role and significance of GSH in cellular redox biology. This includes an update on the formation of GSH-S conjugates or GSH adducts with emphasis given to the mechanism of reaction, the dependence on GST (GSH S-transferase), where this conjugation occurs in tissues, and its significance. The uncovering of the GSH adducts’ formation enhances our knowledge of the human metabolome. GSH–hematin adducts were recently shown to have been formed spontaneously in multiples isomers at hemolysates, leading to structural destabilization of the endogenous toxin, hematin (free heme), which is derived from the released hemoglobin. Moreover, hemin (the form of oxidized heme) has been found to act through the Kelch-like ECH associated protein 1 (Keap1)–nuclear factor erythroid 2-related factor-2 (Nrf2) signaling pathway as an epigenetic modulator of GSH metabolism. Last but not least, the implications of the genetic defects in GSH metabolism, recorded in hemolytic syndromes, cancer and other pathologies, are presented and discussed under the framework of conceptualizing that GSH S-conjugates could be regarded as signatures of the cellular metabolism in the diseased state.

## 1. Structure, Biosynthesis of GSH and Key Enzymes Involved in GSH Homeostasis

Glutathione (GSH) (PubChem CID:124886) is a ubiquitous tripeptide (of L-gamma-Glutamyl Acid-L-Cysteinyl-glycine), which is found in all aerobic organisms. In mammals, GSH accounts for >90% of non-protein thiols. GSH was discovered in 1888 by De-Rey Pailhade in different sources, like blood, sheep brain, fish, and vegetables, and named “philothion” (a substance that loves sulfur) [1,2]. It is highly important to note that this work had been prepared before the peptide bond had been formally proposed [3]. Thirty-three years later, philothion was renamed glutathione by Hopkins and Hunter, who discovered its non-enzymatic reducing power, water solubility, and its high reactivity with dyes and several other chemicals [4,5,6]. Meister, who contributed a great deal to elucidating the chemical structure of GSH, wrote a detailed historical review [7]. Thereafter, the research on GSH homeostasis was extrapolated, and nowadays, there are nearly 59,000 retrievals in PubMed (research articles, reviews, and others) referenced under the term “GSH”. 

The main organs involved in the homeostasis of GSH are the liver, kidneys, lungs, and skeletal muscle [8]. In addition, red blood cells (RBCs) are a rich source of antioxidants (mainly GSH and ascorbic acid), counterbalancing the potential harmful effect associated with hemoglobin carrying [9]. Structurally, GSH possesses a gamma–peptide bond, employing the side chain of carbonyl from glutamic acid, rendering GSH resistant to peptidases. The functional group of GSH is the thiol of the cysteinyl moiety. Due to its prominent role in tissues, GSH is found in several forms, with its reduced form the main one. A small quantity of GSH is oxidized, reacting with cellular reactive oxygen species (ROS), yielding GSSG (disulfide GSH) (normal ratio of GSH/GSSG around 100:1). However, GSH is continually being regenerated by GSSG reductase, a mainly cytosolic but also mitochondrial and nuclear enzyme, using NAPDH as a co-factor [10]. The subcellular localization of GSSG reductase coincides with organelles that accumulate high levels of ROS, such as mitochondria, where mainly superoxide radicals are produced [11]. In eukaryotes, the use of alternative initiation codons deciphers whether the produced GSSG reductase will be cytosolic or mitochondrial [12]. A thiol reductase also exists in lysosomes but lacks the specificity of GSSG reductase [13]. In fact, this specificity is exploited for accurate experimental measurements of GSH content [14]. Moreover, there are the mixed disulfides of GSH with protein thiols, which form a major part of total hepatic GSH, as uncovered using GSH-tagged mono-bromobimane [15]. Last but not least, GSH has been found in the form of the covalent GSH S-conjugates of both xenobiotics and endogenous substances, incrementally increasing the human metabolome library. A detailed presentation of these GSH-centered metabolites takes place in Section 3. 

GSH is biosynthesized through two sequential ATP-dependent enzymatic reactions. At first, glutamic acid is bonded via gamma–peptide linkage to cysteine, which is catalyzed by gamma-glutamyl-cysteine synthetase or ligase (GCS or GCL). GCL is the rate-limiting enzyme in the biosynthesis of GSH, being competitively inhibited by GSH itself [16]. Among glutamic acid, cysteine, and glycine, cysteine is found at a lower concentration in plasma and tissues, and cysteine is thus the rate-limiting substrate in GSH biosynthesis. The GCL enzyme is a heterodimer composed of a catalytic and a regulatory subunit. Buthionine sulfoximide (BSO), the gold standard in the experimental depletion of GSH, inhibits this enzyme by forming transitional state analogs [17]. The second raw enzyme is GSH synthetase (GSS), condensing the gamma-glutamyl-cysteine moiety with glycine, via a regular peptide bond. Both enzymes are characterized by a low Km for ATP (at low μM level) [18], thus permitting GSH biosynthesis to continue in hypoxic and/or energy-depleted conditions, and highlighting its significance for body homeostasis. Cysteine is supplied to the organism via nutrition, protein catabolism and via the cystathionine pathway, acting in the liver to synthesize cysteine from methionine [19]. However, in extracellular fluids, such as plasma, cysteine is easily oxidized in the dipeptide cystine. Both the enzymes of GSH biosynthesis are expressed in multiple tissues, such as liver, kidney, muscle, brain, and RBCs [8]. In fact, the gene encoding the catalytic subunit of GCL (*GCLC*) is under the control of the antioxidant-responsive element (ARE), promoting the binding of the major detoxification-involved transcription factor (TF), nuclear factor erythroid 2-related factor 2 (Nrf2) [20]. To this end, multiple factors, such as H_2_O_2_, ionizing radiation, smoke, and chemotherapeutics, induce the transcription of *GCLC* [8].

Free heme is known to be a major, intrinsic oxidant promoting severe damage to nearby tissues when released under hemolytic conditions [21,22]. Hemin (an oxidized, experimental form of heme) has been recently found, by our research group, to stabilize the Nrf2 TF via action at the protein level without affecting its mRNA levels in pro-erythroid K562 cells [23]. Mechanistically, hemin leads to inhibition of the Kelch-like ECH-associated protein 1 (Keap1)-dependent ubiquitination and degradation of Nrf2. At the same time, Keap1 becomes the substrate of the E3 ligase, attached to the holo-complex of Keap1-Nrf2 [24], and leads to the ubiquitination and proteasomal degradation of Keap1. Nrf2 is, thus, free to translocate into the nucleus and induces, by activating the transcription of GSH biosynthesis-related genes, *GCLC* and the gene of cystine glutamate antiporter (*xCT*), along with the gene of glutathione S-transferase P1 (*GSTP1*), to a lesser degree. The net result recorded is an increase in GSH content associated with the cytotoxic actions of hemin. There is indeed a discrepancy here, since the pro-oxidant effect of hemin diminishes GSH at the intracellular level in both RBCs and astrocytes [25,26]. In this cell model, an assumption would be that the concomitant induction of the *xCT* leads to a continuous supply of cystine, thus replenishing GSH.

The plasma concentration of GSH has been proposed as an indicator of the body’s health status [27]. Plasma GSH (calculated at 3.6 µM) is representative of total GSH [28]. This plasma concentration is at least 10-fold lower that the concentration of GSH found in the tissues. RBCs’ concentration in GSH is among the higher, at 0.4 to 3.0 mM, characterized by interindividual variations [29]. Liver concentration is the higher in the body, reaching 10 mM [30]. Lungs are charged with the role to detoxify air pollutants and ROS and thus contain 10-fold more GSH than plasma (200–400 μM) [31]. In the brain, the GSH concentrations reach 8 mM, as measured in astrocytes [32] (see [33] for a detailed analysis of organs’ concentration in GSH). Plasma GSH comes from the liver, skeletal muscle, and RBCs’ excretion [34]. To this end, it has become clear that GSH has to be transported not only between the different organs but also from the cytoplasm being synthesized to mitochondria, the endoplasmic reticulum [35], and nucleus. There are indeed transporters of GSH, which are mainly members of the multidrug-associated resistance protein (MRP) family of ABC transporters and also of transporters named organic anion transporting polypeptides (OATPs) [36,37]. In mitochondria, porins are exploited for the transport of GSH [10] as well as the glyoxylases’ system [38]. Half of the quantity of GSH exported by the liver is driven to bile (GSH at 8–10 mM) [39] via mainly MRPs. 

**Figure 1:**
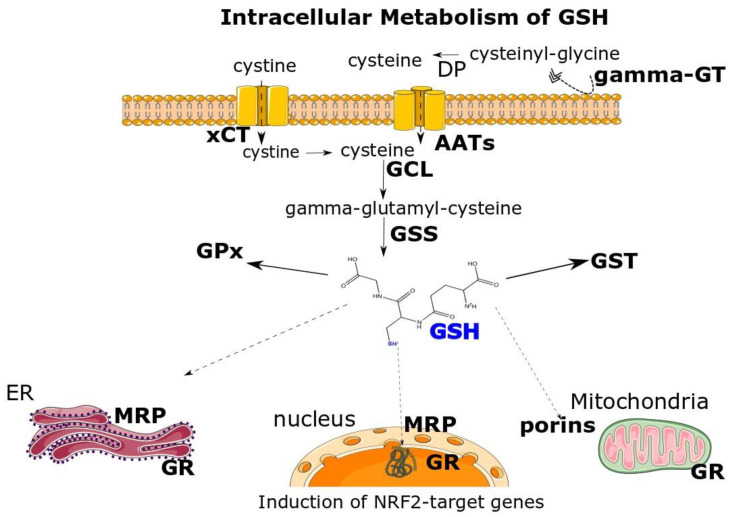
Multifaceted intracellular metabolism of GSH. Cysteine, as the rate-limiting substrate for GSH’s biosynthesis, can be supplied to cells indirectly by the cystine/glutamate antiporter (xCT). Subsequently, the intracellularly imported cystine is reduced to cysteine. Moreover, GSH can itself supply cysteine by the sequential action of extracellular gamma-glutamyltranspeptidase (gamma-GT) and dipeptidases (DP) releasing cysteine that is transported inside cells by common amino-acid transporters (AATs). Glutamate cysteine ligase (GCL) and GSH synthetase (GSS) act in the cytosol to synthesize GSH, serving the coenzyme to glutathione S-transferases (GSTs) and glutathione peroxidase (GPx). GSH is transported in the endoplasmic reticulum (ER), nucleus and mitochondria via multidrug-associated resistance protein (MRP) and porins, respectively. In addition, GSSG reductase (GR) regenerates the GSH pool. Different oxidants, xenobiotics and anti-oxidative compounds induce the GSH biosynthesis, via the NRF2 transcription factor, by upregulating *GCL* and/or *xCT*. Parts of the figure have been acquired by Smart Servier Medical Art.

GSH homeostasis is thus a complicated process depending on the biosynthesis, transport, and utilization (as at GSH-S conjugates) as well as catabolism and regeneration of GSH (Figure 1). The membranous enzyme gamma-glutamyl transpeptidase (gamma-GT) bound in liver and bile ducts catabolizes the gamma–peptide bond of GSH, releasing the cysteinyl–glycine dipeptide cleaved by normal peptidases [40]. In fact, this is the major pathway used also for the catabolism of GSH S-conjugates, as discussed at Section 3. Regarding the key enzymes involved in the metabolism of GSH, there are the GSH peroxidases (GPx), involved in ROS detoxification (Section 2), and GSTs, implicated in the covalent conjunction of GSH to mainly detoxify toxic compounds (Section 3). 

## 2. The Biological Functions of GSH

**Figure 2:**
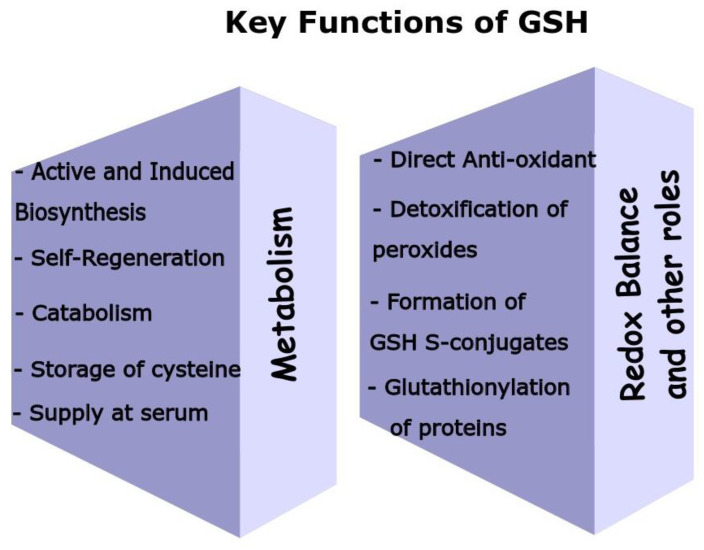
GSH employs multiple biochemical pathways to support key roles in mammalian cells and maintain the redox cycle. By taking into account all these functions and enzymes involved, GSH is indeed a key metabolite. GSH scavenges various ROS either directly or via the action of GSH peroxidases, where GSH is a co-factor. Moreover, GSH serves as a stable storage of the sulfhydryl group, while the self-regenerative capacity of GSH, by the GSH reductase, contributes to maintain its cellular levels. GSH biosynthesis occurs at high rates at many organs induced by oxidative stress or other stimuli. RBCs, liver, and skeletal muckle support plasma GSH’s levels, while liver (via the gamma-GT) is able to accelerate the uptake of GSH’s precursors. Finally, GSH forms various GSH S-conjugates with many different structurally compounds acting as the substrates, which is an event of vast importance in cell protection.

GSH is indeed contained inside cells in concentrations higher than most metabolites [41]. It is not an exaggeration to say that GSH possesses multiple and highly crucial functions in the body [42] (summarized in Figure 2). 

### 2.1. Antioxidant Role

The antioxidant property of GSH is probably the most known of its multilevel actions [43]. GSH is able to directly scavenge several ROS (hydrogen peroxide, superoxide and others) [44] and RNS (as peroxynitrite) [45]. GSH, reacting in vitro even at very low concentrations (10 μM), scavenges these reactive species, as evidenced by electron paramagnetic resonance spectrometry [46], and it decomposes hydrogen peroxide [47]. The pKa of the thiol moiety of GSH is at 9.6, providing a low reactivity [48], but this is not the only parameter that counts (see Eh below). In these reactions, GSH is converted to intermediate products like glutathiyl radicals and sulfenic acids. A detailed review regarding the reactions’ kinetics can be found elsewhere [49]. Given the relative intracellular quantities of these oxidants at the nM level, even under oxidative stress, it can be drawn than GSH can efficiently react with them [50]. This powerful antioxidant action of GSH led to the conclusion that it is the main mechanism of the cytoprotective action of the N-acetyl-cysteine (NAC), a rather weak antioxidant, acting as GSH’s precursor [43,46]. GSH can form a barrier to intracellular ROS propagation and be an important gatekeeper of mitochondrial DNA integrity [51], as shown in human lymphocytes [52]. Moreover, during the phase I detoxification reaction in liver cytochromes P450 enzymes, ROS are produced, such as in alcohol-induced liver damage [53]. 

GSH serves as a co-factor of the antioxidant-related enzymes, the glutathione peroxidases (GPxs) [49]. These enzymes accelerate the antioxidant potential of GSH, detoxifying mainly peroxides including, but not limited to, hydrogen peroxide. Eight different isoenzymes of GPxs exist, differing in their localization and specificity for phospholipid peroxides, cholesterol peroxides and others. Toxic peroxides are converted to non-toxic alcohols in a process coupled to GSH dimerization [54]. In addition, GSH aids in the regeneration of both vitamin C and E, being oxidized via reacting with ROS and lipid peroxides [55]. The advantage that GSH possesses is its enzymatic recycling by GSSG reductase, as analyzed in Section 1.

### 2.2. Cell Redox Status

In starting to analyze the other key roles of GSH, one should consider that GSH is the safe body’s alternative for storage of the unstable cysteine. Once needed, GSH will be degraded by the gamma-glutamyltranspeptidase (gamma-GT). The gamma-glutamyl cycle acts in organs, like the liver and kidneys, as a carrier transport system for amino acids [56]. Intracellularly, GSH has the prominent role of acting as the redox buffer to protect the proper folding of proteins. Cysteines are the highly conserved residues in proteins that are implicated in key functions, such as catalysis and metal binding [57]. Upon the deprotonation of cysteine to the thiolate ion, it is vulnerable to oxidation and adduction by electrophiles, disrupting the proper folding and function. For instance, a hallmark in hemin-induced cytotoxicity (HIC) is the perturbation of proper folding of proteins [58] and the abnormal cross-linking of RBCs’ major cytoskeletal proteins [59]. GSH, in such a high intracellular concentration, contributes to preventing these abnormal effects. To gain further insight into the high reactivity of thiols, it is worth considering Keap1, which acts as a sensor for electrophiles through its highly reactive cysteines, bearing a suitable environment to lower the pKa [60]. This binding leads to the inhibition of the Keap1-dependent ubiquitination of Nrf2 TF, stabilizing it, to induce on time a battery of cytoprotective genes. 

The GSH/GSSG along with the couples of NADPH/NADP^+^ and thioredoxin (Trx)-SH/Trx-SS are essential in maintaining the cellular redox state [61]. In vivo, the redox potential of the GSH/GSSG couple (*E*_h_, as calculated by Nernst equation [62]) is between −260 and−150 mV in different tissues [63]; the more negative the Eh value, the more potent the reductant. Under mild oxidative conditions, cellular signaling is affected, as key TFs, Nrf2, Nuclear Factor-kB, and Activator Protein (AP-1) are redox dependent [64]. Upon oxidative stress, the GSH/GSSG ratio significantly decreases, since GSH is consumed due to covalent conjugation to electrophiles, the formation of mixed disulfides with proteins, and the export of GSH and GSSG to reach places with high oxidative milieu [65].

The covalent conjugation of GSH to cysteinyl residues of proteins is an important post-translational modification (S-glutathionylation) [66]. One of its roles is the protection of this critical residue from oxidative stress-induced damage. Moreover, this modification has a regulatory roles in the function of proteins, as in the case of Heat Shock Protein 70 (HSP70), where the S-glutathionylated form mimics a substrate-binding condition [67], or in glyceraldeyde-3 phosphate dehydrogenase (GAPDH) where the GSH-mixed enzyme is enzymatically inactive [68]. S-glutathionylation is supposed to occur directly by GSH, but it is also accelerated by the enzymatic action of GSTs and at the same time be reversible by glutaredoxins. 

One should not forget to mention that GSH is an important antioxidant keeping the redox balance in the endoplasmic reticulum (ER) [69]. As evidenced by the pioneering work of Hwang et al., using a small, radiolabeled peptide, susceptible to oxidation, the GSH/GSSG ratio in ER is remarkably low, ranging from 1:1 to 3:1. This oxidative environment is permissive for the action of protein disulfide isomerases (PDIs) toward the correct folding of proteins. To this end, the concentration of ER in GSH was significantly decreased by the expression of a chimeric γ-transpeptidase, while several proteins, used as the control, still properly folded [70]. 

### 2.3. Formation of GSH S-Conjugates

The conjugation of GSH to toxic electrophilic compounds is an underestimated function of GSH, taking into account its merit. As eloquently pointed out by Pizzorno, GSH does not merely scavenge ROS produced by the harmful oxidant but deals with the “problem” itself [41]. An example will be given with HIC, where a molar extracellular, excess of GSH (or other thiol, such as NAC and cysteine) conjugates with hemin, leading to a significant decrease in hemin’s intracellular accumulation [71,72]. To this end, ROS accumulation, impaired proteostasis, cell-cycle arrest, and non-classical apoptotic cell death, all molecular signs of HIC [23], were withdrawn in a dose-dependent manner proportional to the thiols’ quantity. 

In fact, it has been long known that conjugation reactions are Phase II biotransformation reactions that occur mainly in the liver. The addition of GSH moiety takes place primarily by GSTs decreasing both the hydrophobicity and toxicity as well as promoting the detoxification and elimination of the unwanted compounds [73]. In some cases, the conjugation renders the resulting metabolites chemically inert or destabilized. There is also the case that GSTs are recruited for the biosynthesis of biologically important molecules, such as in leukotrienes. The substrates for these reactions are both endogenous (intrinsic) and exogenous (xenobiotics), being mainly electrophiles, Michael acceptors, epoxides, electron-withdrawing structures and carbonyls, which are able to react through nucleophilic additions with GSH. Moreover, nucleophilic substitutions and displacements are also important conjugation reactions. Over the years, many such novel metabolites were uncovered, referred to as GSH S-conjugates or GSH adducts, which were assigned their name by the chemical entity adducted. 

Multiple families and classes of GSTs exist in mammals, including soluble (alpha, mu, pi, theta, zeta, omega, sigma and kappa) and six-membered membranous (microsomal) named membrane-associated proteins in eicosanoid and glutathione metabolism (MAPEG), which all have a well-conserved G-(Glutathione-binding) site. An important step in the catalysis is the activation of GSH to the thiyl radical. A critical residue (tyrosine, or serine, or cysteine) is responsible for GSH activation in the G-site [74]. The H-site composed of mainly hydrophobic residues is responsible for the substrate binding [75]. Overlapping substrates, serial reactions of nucleophilic addition, isomerization, and the stabilization of transitions states are common between the different GSTs [54,76]. 

## 3. GSH Conjugates: Mechanisms and Biological Significance

### 3.1. Extrinsic Compounds (Xenobiotics)—The Case of Poisons/Toxins

Benzenes are found almost everywhere in our daily life (in plasticizers, detergents, dyes, etc.). In the 18th century, it was discovered that benzenes are metabolized through the action of CYPs to epoxides and dihydrodiols [77]. These aromatic hydrocarbons are associated with chronic toxicity problems: mainly hepatoxicity, myelosuppression and carcinogenicity. In general, through a cascade of metabolizing reactions, at least two separate classes of GSH S-conjugates are produced. Interestingly, this case of adducts is an exception to the general rule dictating the GSH-S conjugation leading to detoxification. Cytotoxic actions had been assigned to members of this class of adducts.

The initial step in the metabolism of benzenes is the formation of benzene oxides. Bromobenzene is a very common toxic pollutant that is used in the chemical industry [78]. Its main metabolite is the hepatotoxic 3,4-bromobenzene oxide [79]. As shown by using [^14^C]-bromobenzene, the phase I bioconversion (primarily by CYP2E1) of bromobenzene to 3,4-bromobenzene oxide occurs not only in the liver but also in both bronchiolar and alveolar cells of the lungs [80]. By the enzymatic action of microsomal GSTs, the 3,4-bromobenzene oxide yields the bromobenzene–GSH adducts [81]. These isomers of bromobenzene–GSH adducts are formed due to the addition reaction of GSH to the epoxide chemical group (Table 1). 

The remnant quantity of benzene oxide, not being conjugated with GSH, is converted to phenol, which is a highly toxic compound [82]. These steps are catalyzed by both epoxide hydroxylases and are also spontaneous. Phenol can be detoxified with glucuronic and sulfate but also via the conversion to 1,4-benzoquinones; it reacts with GSH, giving rise to multiple GSH adducts. The first adduct in the row, hydroquinone–GSH conjugate (GS-HQ), is not chemically stable, participating in a cascade of reactions and yielding multiple GSH-S adducts, such as GSH-1,4 benzoquinone and 2,5-GSH-1,4 hydroquinone. As such, the formation of the hydroquinone–GSH conjugate cannot be considered as a detoxification mechanism itself, causing the resulting adducts to be nephrotoxic and myelotoxic. In particular, upon administration in mice, only the mono-glutathionylated adducts were not toxic to kidneys, while a correlation was recorded between the number of glutathione substitutions and the nephrotoxicity observed [83]. Hydroquinone administration was correlated with a high level of accumulation of its degradation products (see Section 3.4 for the excretion of GSH-S conjugates) in urine and bile, highlighting a mechanism for the nephrotoxicity and nephrocarcinogenicity detected [84]. When hydroquinone and phenol were administered in mice, the hydroquinone–GSH conjugates were detected in high quantities in the bone marrow [85]. The administration of 2,6-bis(glutathion-*S*-yl) hydroquinone in mice led to significantly less hemoglobin production in reticulocytes, as ascertained by [^59^Fe] incorporation. The authors concluded that these metabolites of benzene, despite being accumulated in vivo at low levels, constitute a major part of benzene-related hematotoxicity. 

Naphthalene, another benzene compound, is contained in mothballs and associated with hemolysis, acute kidney and liver injury. NAC, ascorbic acid and methylene blue are used as antidotes in the clinical practice. The exposure of mice to naphthalene, through the respiratory track, causes necrotic death in lungs, which is associated with a depletion in GSH levels [86]. Its epoxide metabolites are considered highly reactive and form conjugates with GSH in the presence of GST [87,88].

Quinones are a class of compounds with diversified functions derived by aromatic compounds from the conversion of an even number of double bonds to carbonyls. GSH was found to react with many such compounds, while the biological result of the conjugation varies, as discussed below. Bisphenol A (BPA) is a frequently found quinone that is contained in plastics. BPA’s metabolites are genotoxic, reacting and depurinating guanosine and adenosine [89]. Moreover, BPA intervenes with estrogen signaling via its binding to estrogen receptors a and b [90]. GSH forms adducts with its metabolite, bisphenol A-3,4-quinone (BPAQ), yielding two different products possessing one or two molecules of GSH [91,92]. Multiple metabolites are produced by CYPs through ipso substitution mechanisms; all these compounds react with GSH [93], while it is not known yet whether the BPAQ-GSH adducts detoxify or could increase the cytotoxicity of BPA and its analogs. Polychlorinated biphenyl (PCB) quinones are also environmental pollutants. PCBs via the action of CYPs are converted into the active metabolites [94]. The Michael addition of GSH displaces the chlorine on the quinone ring. To the same frame, GSH adducts to benzoquinone form the 2-S-glutathionyl-1,4-benzosemiquinone radical inside rat hepatocytes [95]. Quercetin o-quinone derives from the autoxidation of catechol-containing antioxidants, imposing possible toxicological risks [96]. These unstable metabolites react with GSH in the A ring of quercetin at multiple sites. The resulting isomers, the 6-glutathionyl-quercetin adduct and 8-glutathionyl-quercetin adduct, are able to isomerize spontaneously. These quercetin–GSH conjugates were found to be excreted by human aortic endothelial cells via induced MDR exporters [97].

Aflatoxins are toxins produced by toxigenic fungi correlated with hepatotoxicity and hepatocarcinogenicity. GSH conjugates to their epoxides protect from the formation of adducts of aflatoxin to DNA. The aflatoxin–GSH adducts are formed in the liver and excreted to urine, which is considered a major protective mechanism against the detrimental reactions of aflatoxins with biological macromolecules [98]. In fact, the *GSTPA3* is under the transcriptional control of Nrf2, and its induction protects the mouse liver from the hepatocarcinogenicity induced by Aflatoxin B1 [99]. Formaldehyde forms various adducts with GSH, among them a cyclic adduct, BiGF 2 ((2S,7R)-7-(carboxymethylcarbamoyl)-5-oxo-9-thia-1,6-diaza-bicyclo [4.4.1] undecane-2-carboxylic acid), containing two equivalents of formaldehyde and one molecule of GSH. This adduct has potent biological significance, as both the reactivity and toxicity of formaldehyde are being masked. This reaction impedes the involvement of both the thiol group and the γ-glutamyl-amine of GSH and proceeds in pH levels of plasma [100]. Isothiocyanates, such as allyl isothiocyanate and sulforaphane, are natural preventive agents to carcinogenesis, acting, among other mechanisms, via the activation of the Nrf2 TF [101]. However, in concentrations higher than those acquired by food consumption, some members of isothiocyanates act as pro-carcinogens. Isothiocyanates form adducts with GSH, but when these adducts are added in culture, the allyl isothiocyanate conjugates are as cytotoxic as the parent chemical, while the GSH-benzyl isothiocyanates are even more cytotoxic. Excess GSH diminishes the toxicity of the conjugates, leading to the conclusion that the mechanism of their toxicity depends on the instability of these conjugates, releasing the isothiocyanates [102]. 

**Table 1:** Presentation of GSH S-conjugates with toxic xenobiotic compounds.

Xenobiotic Compound (Reactant)	GSH-S Conjugate (Product)	Chemical Group(s) Involved	Tissues/Organ	Biological Significance	Spontaneous (Direct) and/or GST-Dependent
Benzenes and their Metabolites					
3,4-Bromobenzene oxide [79]	Bromobenzene–GSH adducts (4-glutathionyl conjugate, 3-glutathionyl conjugate)	Epoxide	Liver	Mainly bromobenzene detoxification	GST
1,4 Benzoquinone	Hydroquinone–GSH conjugates	C-C double bond	Liver, bone marrow	Not detoxification; they are labile to a cycle of formation of myelotoxic, multiple GSH S-adducts	Spontaneous
Naphthalene [86]	Glutathionyl–naphtalene adduct	Epoxide	Liver, lung, nasal mucosa	Probably detoxification	Spontaneous, GST [87]
**Quinones**					
Bisphenol A-3,4-quinone (BPAQ) [91]	BPAQ–GSH adduct	C-C double bond	Liver [93]	Not known yet (detoxification of BPAQ or more toxic)	Spontaneous
Polychlorinated biphenyl (PCB) quinones [94]	Glutathionylated conjugated hydroquinone	C-C double bond	Liver	As for BPA	Spontaneous
Quercetin o-quinone [96]	Glutathionyl–quercetin adducts	C-C double bond	Liver [103], aortic endothelial cells [97]	Possible protective role, as these conjugates are excreted from cells [97]	Spontaneous
**Other chemical groups**					
Aflatoxin	Aflatoxin B1(AFB1)–GSH conjugates	Epoxide	Kidneys, liver	Detoxification [98]	GST
Isothiocyanates	Isothiocyanate–GSH adduct	Isothiocyanate group	Liver, kidney [104]	Unstable conjugates, release isothiocyanates [102]	GST, spontaneous
Formaldehyde	BiGF 2	Carbonyl	Liver and possibly in other tissues	Masking, detoxification [100]	Spontaneous

### 3.2. Extrinsic Compounds (Xenobiotics)—The Case of Medicines (Drugs)

Drugs are commonly conjugated with GSH, leading mainly to their excretion out the body (Table 2). Acetaminophen (APAP) toxicity, due to overdose, affects a large number of people in modern societies. The main toxicity provoked is acute liver damage, which is mechanistically provoked by the aberrant adduction of the toxic metabolite N-acetyl p-benzoquinone imine (NAPQI) to cysteinyl residues of key liver proteins [105]. The hepatoxicity is severe and irreversible if left untreated after a while, and the administration of NAC is the gold standard antidote. Backs in 1970s, Mitchell et al. published several back-to-back research works about the mechanism of APAP-induced hepatotoxicity. By exposing mice to phenobarbital, an inducer of hepatic metabolism, the researchers recorded increased liver damage, responding to APAP [106]. The opposite results were seen by piperonyl butoxide, which is an inhibitor of CYPs P450 metabolism. Research works by different teams highly speculated that the toxic metabolite involved was a potent electrophilic-arylating agent [107]. The NAPQI is a bioactive compound produced by Phase I cytochrome 450 enzymes [108,109]. Moreover, the characteristic of APAP-induced liver damage is the depletion of hepatic GSH. The covalent binding of NAPQI to GSH leads to GSH’s depletion [109]. APAP–GSH conjugates are formed via Michael addition to a NAPQI 2,3-carbon–carbon double bond, while a portion of the reactant is reduced back to APAP via the reaction of GSH to the imine double bond [110]. The reaction has a slow rate of spontaneous kinetics, while it is accelerated by the action of GSTs, being catalyzed by several classes of them, mainly (but not exclusively) hepatic [111,112].

The GSH-mediated detoxification of chemotherapeutic (anticancer) agents needs a special merit, since it is frequently associated with the molecular basis of resistance to the cytotoxic action of these drugs (see [113] for a dedicated review on anticancer drugs–GSH adducts). Cisplatin is a widely used anticancer drug, acting as an alkylating agent, adducting to DNA and inhibiting proliferation of the highly dividing cells [114]. GSH forms adducts via a nucleophilic substitution reaction mechanism to cisplatin (to its intracellular form, platinum (II) aqua form) via a molar ratio of 2:1, as shown by mass spectrometry, both in vitro and inside leukemic cells [115]. Diglutathionyl–platinum adducts facilitated the export of cisplatin by cells, restricting its intracellular accumulation and thus the cytotoxic action. Although the aforementioned mechanism describes the diglutathionyl–platinum adducts as a contributor of the resistance exhibited by cancer cells to cisplatin, there are data supporting the opposite result. These adducts, purified by anion exchange chromatography, are characterized by the same property of the parental compound (cisplatin) into the in vitro inhibition of protein translation. Importantly, these adducts were detected also in multiple organs of *Rattus norvegicus* such as the heart, pancreas, and liver. The tissue-specific differential expression of *GSTP1* was correlated with their pattern of accumulation [116]. In a dedicate research work related to the involvement of GSH in the cancer cells’ resistance (using cell fractionation analysis), the authors concluded that the formation of diglutathionyl–platinum adducts accounts only partially for cancer cells’ resistance [117].

The list of chemotherapeutics forming GSH conjugates is quite lengthy; this includes chlorambucil (adduct formation through nucleophilic substitution of the atom of chlorine [118]), cyclophosphamide (acrolein is one its main metabolites conjugating to GSH and becoming significantly less cytotoxic than the parent compound [119]), mitomycin C (forming mono- and bis-conjugates with GSH to be detoxified [120]), and various tyrosine kinases inhibitors (such as axitinib and sunitinib [121]). 

Landomycin E is a novel, potent antineoplatic drug that is structurally related to adriamycin. It is highly promiscuous, since it is not affected by the MDRs, while its anti-leukemic action depends mainly on mitochondrial damage, inducing apoptosis [122]. Landomycin E forms covalent adducts with GSH, which was proposed as another mechanism of its chemotherapeutic action [123]. 

Azathioprine is an immunosuppressive drug used prophylactically prior to organ transplantation and in autoimmune diseases. This drug is an example of a case where GSH-dependent thiolysis is needed to acquire the active compound, which is 6-mercaptopurine (bioactivation process) [124]. Azathioprine is a masked form of 6-mercaptopurine, since it is composed of 6-mercaptopurin linked via a thioether bond with an imidazole ring [125]. GST-dependent thiolysis results in the release of 6-mercaptopurine [126]. 6-thioguanine triphosphate (6-Thio-GTP) derived from 6-mercaptopurine leads to the mitochondrial-dependent apoptotic death of T-cells via the specific binding and inhibition of the small GTPease, Ras-related C3 botulinum toxin substrate 1 (Rac1) [127]. This inhibitory effect of azathioprine acts on CD28-activated T cells. 

Moving on other drugs being conjugated with GSH, simvastatin is such a case. Simvastatin hydroxyl acid comes from serial hydrolytic reactions of simvastatin, and it is the active metabolite that inhibits the 3-hydroxy-3-methylglutaryl (HMG)–coenzyme A reductase. The 5′,6′-dihydroxy-4′a-glutathione adduct of simvastatin has been identified by mass spectrometry analysis in rat bile [128]. Moreover, its degradation products were detected, suggesting that these GSH thioether adducts act to increase the elimination of simvastatin from the body.

Diazepine, via its electron-rich tricyclic core, forms adducts with GSH [129]. Efforts have been made to reduce this electron-rich source to potentially decrease its metabolism and increase the pharmacological action. Barbital, a sedative agent, accepts multiple modifications through P450 metabolism in the liver, kidneys, adrenal glands, and lungs. 3′-oxohexobarbital was found to react with GSH in vivo (rats, guinea pigs) and forms an unstable intermediate thioether adduct, which segregates into 1,5-dimethylbarbituric acid and a cyclohexanone–glutathione adduct [130]. Importantly, the former metabolite is detected in urine and the latter is detected in bile, depicting that GSH conjugation highly probably facilitates the elimination of barbital from the body. Chronic poisoning due to arsenic correlates with an increased predisposal to cancer for different organs [131]. Several GSH–arsenic conjugates were detected in both bile and urine, implying these metabolites have detoxifying properties [132]. On other hand, arsenic-based compounds, with arsenic trioxide as the prototype, have already been approved by the FDA as a gold standard differentiation therapy to acute promyelocytic leukemia (APL) that is refractory to conventional therapies [133]. The formation of GSH conjugates to phenyl arsine oxide (another arsenic-based drug) results in the inhibition of GSTP1 action [134], highlighting another example of cellular signaling by GSH S-conjugates.

Table 2 illustrates representatives of common drugs forming adducts with GSH. The list actually contains many more such compounds. Notably, independent research groups attempted holistic approaches to discover novel drug–GSH adducts [135,136,137]. In particular, different biological sources (human liver microsomes, liver S9 (microsomal and cytosolic) fractions, and human liver hepatocytes) were tested for their ability to form GSH-S conjugates with 10 commonly prescribed drugs [135]. Among these compounds, there were ones known to provoke hepatoxicity and/or hepatocarcinogenicity. Moreover, three GSH S-adducts (with clozapine, ticlopidine, and diclofenac) are already known. The antiplatelet ticlopidine [138] is affected in extended Phase I metabolism, which is correlated with its bioactivation, giving rise to multiple GSH S-adducts. Thus, in the GSH scanning approach, compounds such as dichlorofenac, but not chloramphenicol and caffeine were found to form GSH-S adducts. Derivatives of the GSH S-adducts such as S-cysteine, S-cysteinylglycine, and N-acetyl-cysteine were also detected [135]. There is also the case of addictive drugs: for example, cocaine is known to decrease the liver’s GSH content and has been found to be conjugated with GSH, highlighting that cocaine impairment of protein thiols can be a molecular mechanism of its toxicity [139]. Sixteen addictive drugs or drugs under the general category of antidepressants were screened during an in vitro GSH trapping method (in the absence of GST, but with 2 mM GSH, in a ratio drug:GSH of 1:2–1:20) and analyzed by quadrupole time-of-flight (QTOF) MS/MS analysis [136]. The MS data were screened for patterns of daughter ions, revealing 10 out of 16 compounds as adducts with GSH (Naltrexone, Diazepam, Oxycodone and others). Implications in the molecular basis of their toxicity were proposed by the authors. 

**Table 2:** Representatives of commonly prescribed drugs conjugated by GSH to being detoxified or bioactivated.

Xenobiotic Compound (Reactant)	GSH-S Conjugate (Product)	Chemical Group(s) Involved	Tissues/Organ	Biological Significance	Spontaneous (Direct) and/or GST-Dependent
Painkillers					
NAPQI [112]	APAP-GSH conjugate	C-C double bond, imine double bond	Main liver but also kidneys and others	Detoxification of APAP	Both
**Anticancer drugs**					
Cisplatin	Diglutathionyl- platinum adducts [115]	Pt-O	Cell extracts (leukemia), liver, lungs, heart, kidneys, brain, pancreas [116]	Quench the action of cisplatin, excretion of cisplatin, the adducts still inhibit translation of mRNAs	Spontaneous [115]
Landomycin E [123]	Landomycin E-GSH adduct	C-C double bond	In vitro (cell extracts)	Potent anti-neoplasmatic action for GSH depletion	Spontaneous
**Immunosuppressive**					
Azathioprine [126]	6-mercaptopurine	Thiolysis	Liver	Bioactivation	GSTA [140]
**Cardiovascular disease drugs**					
Simvastatin	5′,6′-dihydroxy-4′a-glutathione adduct of simvastatin [128]	C-C double bond	Bile	Probable detoxification, since it favors the formation of degradation products excreted in bile	Probably GST-dependent
**Antidepressants**					
Diazepine [129]	GSH–adduct of Tricyclic Diazepine	High electron density of tricyclic core	In vitro	Probable detoxification	Spontaneous
3′-oxohexobarbital [130]	Cyclohexenone–glutathione adduct	C-C double bond	Liver, bile	Detoxification through excretion in bile	Probably by GST
**Anticoagulant**					
Ticlopidine [138]	Multiples ticlopidine–GSH adducts	C-C double bond and epoxide	Liver	Not known, perhaps detoxification	GST, unknown if spontaneous
**Addictive drugs**					
Cocaine [139]	Cocaine–GSH adducts	Aryl moiety	Liver	Not known, possibly detoxification	GST

### 3.3. Endogenous Compounds

There are many types of adducts formed between GSH and divergent endogenous compounds, all of which have key functions (Table 3). In particular, menadione (2-methyl-1,4-naphthoquinone) or vitamin K3 is essential in the body for the synthesis of blood coagulation factors [141]. Much of vitamin K is synthesized from colon bacteria. Inside rats’ hepatocytes, a significant depletion of the GSH pool occurs due to the formation of the GSH–menadione conjugate (2-methyl-3-glutathionyl-1,4-naphthoquinone), which is rapidly excreted out of cells, in the culture medium [142]. At the same time, the treatment with menadione at such high concentrations (50–100 μM) provokes GSH-S glutathionylation to protein thiols. Quinones are known to form adducts with GSH, as discussed at Section 3.1, where quinone rings act as electron-withdrawing structures [143]. Based on the fact that the redox reactive GSH–menadione adducts are excreted, a protective mechanism was proposed to be implicated in catabolic tissues, such as the liver [144,145]. Moreover, it has been found that the rat renal proximal cells metabolize these adducts to cysteinylglycine conjugates [146].

There is a clear correlation between the elevated levels of the hormone 17β-estradiol (E_2_) with the risk of breast cancer [147]. Apart from the receptor-dependent hormone signaling, certain metabolites of estrogens act as direct carcinogens, such as the catechol 4-hydroxyestradiol and its derivative quinones [148]. E2-2,3 and E2-3,4 quinones adduct to DNA, leading to depurination and the accumulation of mutations during replication [149] as well as random losses or gains of metaphase chromosomes [150]. The catechol estrogen quinone–GSH conjugates [151] were detected by HPLC analysis in breast biopsy tissues in patients suffering from breast adenocarcinoma [152]. These adducts are found in significant larger quantities in the tumorigenic samples than in the healthy tissues, while the detection of cysteine and NAC conjugates of the 4-hydroxyestradiol depicted an intratumor metabolic process. Their significance in the mechanism of breast cancer tumorigenesis is highlighted by their proposal as potential biomarkers of its prognosis (discussed in Section 5). 

On the other hand, there is the case of the neurodegenerative Parkinson’s disease, where the catecholamines L-3,4-dihydroxyphenylalanine (L-DOPA) and dopamine oxidize to quinones, producing harmful ROS implicated in neuronal cell death [153]. These quinones’ derivatives form adducts with GSH in a reaction promoted by the presence of iron and copper. The relevant GSH S-adducts and their cysteinyl-metabolites were detected in post-mortem brain samples in patients with Parkinson disease at a low level in all areas of brain but at a relatively high level in the substantia nigra. These adducts reacted with the initial catacholamines quinones, yielding neurotoxic products; one of these products was lethal when administered in mice brain [154]. It is still uncertain whether their formation is implicated in the onset of the disease and related to an increased sensitivity of certain parts of the brain in neurodegeneration or a consequence of the pharmaceutical intervention with L-DOPA.

Eicosanoids, including prostaglandins (PGAs) and leukotrienes, are paracrine/autocrine hormones derived mainly by arachidonic acid [155]. They are found throughout the body (intestine, ovary, blood vessels etc.) and involved in multiple functions, such as hormone secretion, ovulation, aggregation and vasodilation. Many enzymes are involved in their synthesis, such as P450 enzymes, 5-lipoxynease, 12-lipoxygenase, which are located in almost all tissues. The class of eicosanoids frequently reacts with GSH, via the action of GSTs, through Michael addition to the α and β unsaturated ketones, as elegantly reviewed [156]. GSH conjugation to the enolised ketone intermediates of ecosanoids could protect cells from their toxic actions. However, there are also cases in which these GSH adducts retain or have novel biological activities, as discussed below.

The first such adduct was discovered for prostaglandins (PGAs) 1 and 2 in human RBCs, depicting that the formation of the PGA1–GSH adduct was accompanied by altered physicochemical properties [157]. This adduct was characterized by GC-MS later on [158]. The liver and kidneys are the main sites for the catabolism of PGAs; rat liver and lung extracts accelerate the conjugation reaction [159,160]. PGA1–GSH adducts are characterized as unique metabolites based on their biological potency. Leukemia cells excreted out PGA1–GSH adducts. It is important to record that when endogenous GSH levels are chemically depleted, the PGA1-induced inhibition of cell proliferation is moderated [161]. The authors concluded that some of the metabolites of the PGA1–GSH adducts act as cytotoxic agents to leukemia cells. Interestingly, detailed biochemical studies revealed novel properties of PGA1–GSH adducts, such as a higher turnover number (Kcat) for PGA E 9-ketoreductase than its substrate [162] and the inhibition of cAMP efflux in RBCs [163].

The conjugation of GSH to leukotrienes (LTs) is integrated to their biological actions. LTC_4_ synthase (a membranous GST) found in hematopoietic cells synthesizes LTC_4_ (LTA_4_-GSH adduct), which is a precursor of cysteinyl-leukotrienes. These compounds are pro-inflammatory lipid mediators, inducing the contractile activity of the bronchi [164] as well as mucus secretion [165]. The enzymatic removal of glutamic acid and glycine from LTC_4_ gives rise to LTD_4_ and LTE_4_, respectively. All these thioether LTs act through the cysLT1 and cysLT2 receptors [166,167].

There is also the case of 4-hydroxynonetal, which is a highly toxic lipid peroxidation product [168]. These aldehydes have a longer half-life than ROS to exert their catastrophic effects, as evidenced in various pathologies [169,170]. A main mechanism of its detoxification is the GSH conjugation, forming the glutathionyl–hydroxynonenal adduct, which is a cyclic hemiacetal [171]. This adduct is formed in at least four times larger quantity than that of 1,*N*^2^-propano adduct to 2′-deoxyguanosine (a DNA adduct) in THP1 monocytes, highlighting its protective action [172]. The dinitrosyl–diglutathionyl–iron complex is a plasma carrier of NO, and interestingly, it acts both as a competitive [173] and allosteric inhibitor of GSTs. The latter means that the binding of the dinitrosyl–diglutathionyl–iron complex to one subunit of GST makes the binding of it to another subunit less favorable (negative cooperativity), demonstrating that the basal enzymatic activity of GSTs has a protective mechanism [174].

**Table 3:** Intrinsic compounds forming GSH S-adducts.

Endogenous Compound (Reactant)	GSH-S Conjugate (Product)	Chemical Group(s) Involved	Tissues/Organ	Biological Significance	Spontaneous (Direct) and/or GST-Dependent
Vitamins					
Menadione [142]	Menadione-GSH conjugate	C-C double bond	Liver	Detoxification through excretion [142]	Spontaneous [143]
**Hormones**					
Estrogen	catechol estrogen quinone–GSH conjugates [175]	C-C double bond	Liver, breast	Decrease in carcinogenic potential	GST
**Neurotransmitters**					
Dopamine	5-S-glutathionyl-catecholamine conjugates [153]	C-C double bond	Brain	Possibly neurotoxic through interconversions [154]	Spontaneous (in presence of tyrosinase) [153]
**Eicosanoids**					
Prostaglandins	PGA1-GSH adduct [158]	C-C double bond	RBCs, liver	Cytotoxic [161], novel biochemical properties [162,163]	Spontaneous, GST
Leukotrienes	LTA_4_-GSH adduct (LTC_4_)	C-C double bond	Hematopoietic cells	Synthesis [166]	GST- LTC_4_Synthase
4-hydroxynonetal (lipid peroxidation product)	3-glutathionyl-4-hydroxynonanal	C-C double bond [176]	Liver [177]	Detoxification	Spontaneous, GSTA4

### 3.4. Catabolism and Excretion of GSH S-Conjugates

The mercapturic acid pathway is the main route for the catabolism and elimination of GSH S-conjugates. This pathway consists of sequentially acting enzymes (GSTs, gamma-GTs, dipeptidases, cysteine *S*-conjugate *N*-acetyltransferase (NAT8)) as well as the transporters for the renal excretion [178]. The result of these enzymatic reactions is the production of cysteinylglycine conjugates, cysteinyl conjugates and N-acetyl-cysteinyl conjugates. The latter ones are characterized by increased hydrophilicity due to ionization of the carboxylic acid at the pH of urine. As evident above, this catabolic pathway is active in many tissues, such as the kidneys, liver, bile ducts, brain and bone marrow. However, the highest expression of NAT8 is in the endoplasmic reticulum of renal proximal tubular cells [179]. 

Hydroquinone (HQ) administration in mice leads to the excretion in bile of four different GSH S-conjugates and one cysteinyl-adduct (2-(cysteinylglycyl)HQ), while the N-acetyl-cysteinyl-adduct, 2-(N-Acetylcystein-S-yl)HQ, is the only thioether adduct identified in urine [84]. The mercapturic acid of aflatoxin B1 (exo-aflatoxin B1 mercapturate) is detected in rats’ urine and excreted in a dose-dependent manner to aflatoxin’s concentration in plasma [180]. Mercapturic acids, such as the N-acetyl-leukotriene E(4), are high-affinity substrates of several renal transporters, such as the renal organic anion tranporter-1 (Oat1) [181]. Mercapturic acids’ profiling has been recently proposed by Gonçalves-Diaset et al. as a possible source of biomarkers (discussed in Section 5) for respiratory, neurological, and metabolic diseases as well as for cancer [182]. 

The specific activity of gamma-GT is high in the luminal membranes of the bile duct epithelium, favoring the formation of the cysteinylglycine conjugates [183]. The S-conjugates inside the bile duct can be absorbed by the gut to blood circulation and proceed for renal secretion either being metabolized inside the gut lumen [184] or excreted to feces (as found for the sulfuric acid ester of the carcinogen 1-methylpyrene [185]). There is another route for S-conjugates’ metabolism, back in the liver, that leads to the removal of the acetyl group by aminoacylases, converting back the mercapturic acids to cysteinyl adducts [186]. In this case, when the cysteinyl-S adduct contains a strong electron-withdrawing structure, cysteine S-conjugate β-lyases convert it to another chemical compound, comprising the sulfhydryl group attached to the parent electrophile, pyruvate, and ammonium. These products can be excreted after UDP glucuronidation or be bioactivated (become toxic) [187]. Halloalkenes are an example of bioactivated compounds transformed first to *S*-(halomethyl)-glutathione and then converted by S-conjugate β-lyases to thioacylating agents, such as the thioacyl halides [188].

## 4. GSH–Hematin Adducts: Characterization, Mechanism of Formation and Possible Implications in Heme Associated Pathologies

GSH–hematin (ferric hydroxide heme) adducts have been recently characterized and detected as novel metabolites in human RBCs’ lysates (hemolysates) [72]. In this section, we wish to refer to the mechanism of reaction between heme (a rather vital molecule for life [189]), and particular its oxidized forms (hematin and hemin (ferric chloride heme)) and GSH as well as the consequences of this conjugation on the cytotoxic actions of the endogenous, electrophile, free heme. Forty years ago, the reaction of GSH with heme was proposed as an internal safety mechanism of RBCs [25,190,191,192]. Possible implications of the discovery of GSH–hematin adducts in RBCs homeostasis and related pathologies are also presented and discussed. 

As analyzed in Section 1, RBCs contain a high concentration of GSH, and both the cycles of biosynthesis and metabolism are highly active [193]. Model analysis for both GSH synthesis and GSSG export depicted that the negative inhibition feedback loop of GCL by GSH does not play the key role in regulating GSH levels in human erythrocytes. RBCs are the transport “vehicle” of GSH between the liver and kidneys (providing precursors of GSH’s biosynthesis) and organs of net utilization (lungs, brain, heart) [194]. RBCs are a rich source of antioxidants to counterbalance the increased risk of oxidative stress, containing ascorbic acid, vitamin E, peroxiredoxins, superoxide dismutase, thioredoxins, and glutathione peroxidase alongside GSH [195]. GSH has a central role in RBCs’ homeostasis, possessing multiple roles as a coenzyme in GPx activity, preventing the oxidation of –SH groups of hemoglobin, and acting as a direct reducing agent of methemoglobin (Fe^3+^) [196]. Congenital hemolytic anemias attributed to defects in the biosynthesis of GSH (in the genes of GCL, GCS and glutathione reductase) highlight the key role of GSH in RBCs’ homeostasis [197,198]. Their symptoms vary from mild hemolytic anemia to moderate and can also include severe CNS damage. On the other hand, the detoxification of toxins is facilitated inside RBCs via the formation of GSH adducts, such as those of menadione, which are accompanied in their export to plasma [199].

RBCs are highly sensitive to oxidative stress due to the huge quantity of hemoglobin they carry (around 19.7 mM of heme/RBC). During the 120 days of their lifespan, RBCs are charged with carrying oxygen from lungs through the oxyhemoglobin (Fe^2+^) and transferring it to tissues, including those in the brain, which is characterized by a high oxidative milieu [9]. A proportion of hemoglobin (1–3%) is autoxidized to methemoglobin, in which reaction mainly superoxide is produced [200]. In addition, RBCs pass through the narrow sinus wall of the reticuloendothelial system (as in spleen), checking themselves for proper conformation and integrity [201]. 

A dedicated system in RBCs consisting of both cytochrome *b*_5_ reductase and cytochrome *b*_5_ exists to catalyze the reduction of methemoglobin, using NADH as the electron donor [202]. Methemoglobinemias either are caused by exposure to oxidants (as benzocaine) or are hereditary disorders of the gene encoding the cytochrome *b*_5_ reductase of hemoglobin [203]. RBCs strongly depend on glycolysis for the production of NADH. The required NAPDH for the action of GSH reductases, peroxideroxins, and Gpxs is mainly supplied by the pentoze phosphate pathway [204]. The enzyme of glycose-6-phosphate dehydrogenase (G6PDH) has a key role in NAPDH production. G6PDH deficiency is the most common enzymopathy of RBCs [205], causing heterogeneous anemias, ranging from fauvism to acute or severe hemolytic anemias. A complete genetic loss of *G6PDH* is incompatible with life maintenance. 

Hemoglobin has to remain in the redox state, since methemoglobin is unable to carry oxygen but is also prone, through autocatalytic oxidative reactions, to lose heme (iron–protoporphyrin IX) [206]. Heme upon its release from its hemoglobin protein chain has a high affinity for membrane phospholipids, being categorized as a lipid intercalator agent [207,208,209]. Hemin acting on RBCs promotes an irreversible cross-linking of RBCs cytoskeleton proteins [59] and the disturbance of proper protein folding [210] leading to osmotic-dependent RBCs lysis [190]. Into this hemolytic mechanism, both ATP and GSH depletion is implicated. Hemin-induced hemolysis sustains a self-propagating effect to release more heme in the nearby tissues. Free heme is assigned many toxic actions, such as being a pro-oxidant [21,22,211], pro-inflammatory [212,213] and vaso-occlussive agent [214,215] that causes the depletion of NO [216] and oxidation of serum lipoproteins (atheromatosis) [21].

The toxicity of free heme is implicated in the pathology of the various etiologies of hemolytic disorders (hemolytic anemias, hemorrhagic strokes and traumas, malaria, sepsis, ischemia reperfusion injury and others [217]). As a consequence, the reaction of RBCs’ GSH with free heme attracted scientific interest very early. In 1981, a series of detailed physicochemical and biochemical research studies began to investigate the reaction between GSH and heme. GSTp (the main isoform of erythrocytes) [218] has been found to be competitively inhibited by hemin with a low Ki (10^−7^ M), suggesting GST has an active role inside RBCs potentially as a hemin-binding protein [219]. In the same year, Chou and Fitch described how thiols (GSH, cysteine, and mercaptoethanol) prevent hemin-induced hemolysis [190]. In addition, hemin was described as an inhibitor of GSH reductase [220]. Shviro and Shaklai recorded shift changes at the maximum absorbance (380–410, Soret peak) of hemin to be caused by the-SH group of GSH, involving also the iron of hemin [191]. In this work, GSH was proposed as a scavenger of hemin or cytoplasmic trap “fighting” hemin from the inside of RBCs to decrease its affinity for both membranous and cytoskeleton proteins. The degradation of heme and release of iron by the reaction with GSH was first described by Atamna and Ginsburg to occur in membranous bound heme at physiological pH [25]. This reaction was proposed to act as a protective mechanism of sickle cell disease (SCD) RBCs. Atamna and his co-worker discovered a linkage between the enhanced levels of non-heme iron found in the SCD RBCs’ membranes and the reaction between heme and GSH. The resulting products had fluorescent properties but were neither biliverdin nor bilirubin. The protective effect of GSH to hemin-induced hemolysis was proven again, giving more details on how GSH worked to keep hemin occupied [192] and restricted its partition in liposomes [221]. However, both the exact reaction products and the underlying mechanism of reaction have been unknown until recently. 

Starting from our recent data on the biological effects of the reaction of GSH with hemin, it leads to the disintegration of hemin’s moiety, halting the intracellular accumulation of hemin, as shown in pro-erythroid K562 cells [71,72]. Mechanistically, the incubation of hemin with thiols (GSH, NAC, cysteine, mercaptoethanol, and thioglycolic acid) provokes major spectrophotometric changes in hemin despite the presence of low-affinity binders in serum [222]. In particular, a molar excess of thiols (from 10 up to 100-fold) over hemin leads to a shift (by 10–20 nm) of the Soret peak of hemin’s spectrum, which is accompanied by its decline to almost disappearance. These alterations in the spectrum of hemin, characteristic of its tetrapyrrole structure, are evident even at the onset of the incubation. In the contrary, the spectrum of protoporphyrin IX, a chemical moiety consisting of the tetrapyrrole ring lacking the iron atom, remains unaffected by the action of thiols. Ferrozine analysis measuring iron, not bound to hemin [223], depicts around half of hemin to be deteriorated by NAC, at 12 h incubation. However, the remaining quantity of hemin could be enough for its cytotoxic effects on K562 cells [23]. Under these conditions and based on the preventive action of NAC on HIC, alternative mechanisms lead to the abrogation of HIC by NAC. Endogenous GSH is a barrier to HIC, as K562 cells simultaneously treated with BSO (the inhibitor of GCL) and hemin exhibit high levels of cell death [72]. However, the extracellular addition of GSH or NAC is adequate to protect cells from HIC even under the chemically depleted conditions in GSH. The direct interaction of NAC and GSH with hemin had to be further investigated in detail to merit the mechanism(s) of the cytoprotective action [224]. 

To understand the nature of this chemical reaction, electrospray, in tandem mass spectrometry (MS/MS) analysis of the in vitro reaction of either NAC or GSH with hemin in phosphate buffer (pH 7.0) at 37 °C was performed. Novel NAC–hemin adducts and GSH–hemin adducts (1st products) are formed by the nucleophilic addition of thiols both to the ethylene bridges of the tetrapyrrole ring (four sites available) and the double bonds of the side chains of hemin (two sites available). Four isomers of the NAC–hemin adduct and five isomers of the GSH–hemin adduct were detected to elute significantly earlier than hemin, delineating the hydrophobicity of hemin to be compromised. The MS fragmentation spectra of these adducts ascertain the thioether bonding among the daughter anions. The characterization of the thiol–hemin adducts adds mechanistic data for the complementary layer of protection, as predicted by the earlier studies [25,191]. Thiol–hemin adducts are probably less prone to intercalate to the cellular membranes and cause lipid peroxidation. As for the proposed mechanism of reaction, electron translocation in the tetrapyrrole ring system leads to the formation of a third covalent bond between the iron and nitrogen. Thus, the extensive chemical rearrangement justifies the spectrophotometric changes of hemin by thiols, and iron appears to be necessary for the completion of the reaction. Moreover, a higher reactivity of GSH than NAC has been found previously for the BPA-GSH conjugates, which is attributed to the lower pKa of the thiolate of GSH exhibited in neutral pH [91].

Moreover, hydroxy- and keto-thiol–hemin adducts (2nd and 3rd products) are formed in the reaction between thiols and hemin. These products are more hydrophilic than the thiol–hemin adducts, as shown by their retention times in reverse phase C18 column analysis. They derive from the nucleophilic addition of water to the thiol–hemin adducts while a pyrrole ring is being formed, leaving the iron bonded to three nitrogens, in this case. This weakening in iron bonding explains the release of iron by them, which is caused by reducing agents such as GSH or ascorbic acid (contained in ferrozine analysis). Quantitation of the ion peaks chromatographs showed that these interconverted reaction products accumulate early at the course of the reaction, but their quantities decline thereafter. This raises the possibility that they might not be the terminal products of the reactions. The important clue is that free iron is not as cytotoxic as hemin, as shown by incubations with iron (FeCl_2_ and FeCl_3_) that are not toxic in culture (preliminary data at [71]) and by knowing that the structure of hemin itself contributes to its toxicity [225]. The degradation of heme occurs by cleavage of the ethylene bridges by HO-1, the main catabolizing pathway for heme, yielding the tetrapyrrole biliverdin IXa, carbon monoxide and iron. HO-1 determines the stereospecificity to the ethylene bridge through the generation of ferric hydroperoxo species [226]. Enzymatic catabolism at the ethylene bridges by the hepatic NAPDH ferricytochrome oxidoreductase yields dipyrolles (propentdyopent) [227]. Moreover, the non-enzymatic degradation of hemin occurs by H_2_O_2_ (forming propentdyopents) [228] and HOCl [229] (yielding a number of degradation products). As discussed above, the chemistry behind the disintegration of hemin/hematin by thiols is indeed intriguing and relatively uncommon. 

Based on the characteristics of *m*/*z* and fragmentation spectra uncovered for the GSH–hemin adducts, we have decided to search for analogous molecular ions in healthy donor RBCs. Hematin (using the OH as stabilizing ion) was detected in high quantities (as RBCs are known to contain heme, not bound to hemoglobin, at around 20 μM [230]). Traces of the molecular ion of the same *m*/*z* as that of the GSH–hemin adduct were also recorded. The treatment of RBCs with hemin increased this quantity, depicting that GSH in the cytosol of RBCs formed a trap for incoming hemin. GSH–hematin adducts were proposed to be formed via the withdrawal of the respective stabilizing ions yielding the same molecular ion [72] (thus bearing identical fragmentation spectra). When hematin release was accelerated due to the addition of acetonitrile and left to react with GSH (contained in RBCs) at 37 °C, high quantities of the various isomers of the GSH–hematin adduct and their hydroxyl derivatives were detected. 

We tend to believe that new routes are opening up for future studies, since the research in this field will probably fire up again. In particular, there are several open questions that need to be answered: Under which pathophysiological situations are the GSH–hematin adducts and their derivatives formed? Do they have any other biological roles, other than the decomposition of free heme, as cell signaling? What is their fate in the human body? Starting from the last question, the main pathway for heme catabolism is its conversion to biliverdin by HO-1 and then by NAD(P)H biliverdin reductase to bilirubin, which is followed by conjugation with glucuronide (mono and diglucoronides). The latter conjugates are excreted to bile and then remain in the large intestine, where the metabolism by flora removes glucuronides and yields urobilinogen and stercobilinogen, which is found in urine and feces. About 250–300 mg of bilirubin is produced per day, while this quantity significantly increases in hemolytic anemias [231]. Interestingly, antioxidative properties have been assigned to bilirubin, since it is able to scavenge peroxyl radicals better than a-tocopherol at the μM scale [232]. However, bilirubin is also neurotoxic at such high concentrations through mechanisms involving lipid peroxidation, as is for the case of hemin [233]. Future studies can prove whether the mercapturic acid pathway is used for the excretion of GSH–hematin adducts or not, in the case that these adducts are indeed formed inside the body. Regarding their potential signaling roles, separation methods, such as those employed previously [116], will shed light on this case. Erythrocyte GST (eGST), GSTP1-1, is over-expressed in nephropathic patients and in healthy subjects living in highly polluted areas [234,235]. We do not know whether the reaction of GSH with free heme is accelerated inside RBCs by the catalytic action of eGST. 

In a recent work published on August 2023 employing *HO-1* knock-out mice, the administration of simvastatin along with angiotensin II provoked renal injury due to accelerated rabdomyolysis [236]. In those animals that survived, the high deposition of heme to kidneys was accompanied by an upregulated metabolism of GSH as an alternative pathway to defend. Although GSH–hematin adducts have not been detected in vivo, the authors proposed the formation of these metabolites in mice kidneys. 

RBCs normally contain a high concentration of bioavailable or free heme, as shown by dialysis experiments [230], derived by the auto-oxidation of 300 million molecules of hemoglobin in each RBC. Thus, we propose that the GSH–hematin adducts’ formation is a protective mechanism of RBCs from the auto-oxidation of hemoglobin to prolong their survival. Under this frame of thinking, we can speculate that in disorders with elevated hematin release, the content of GSH–hematin adducts increases accordingly. Thus, upcoming studies at in vivo conditions can be highly enlightening. In hemoglobinoathies (mainly SCD and thalassemias, but not limited to), the hemoglobin S and globin dimers are prone to lose heme through the formation of unstable hemichromes [237]. In particular, unstable hemoglobins autoxidize and precipitate, forming the hemichromes, containing all forms of hemoglobins and leading to the release of heme [238,239]. The metabolomic analysis of RBCs from 28 SCDs patients reveals significant fluctuations in 31 metabolites compared to normal RBCs; among them, the GSH precursors are found elevated while a decrease in GSH characterizes the pathogenic RBCs [240]. Moreover, the extent of autologous IgG binding (a sign of aged RBCs), promoting the clearance of RBCs, correlates with their content of fluorescent heme degradation products [211]. As such, due to heme leakage during the aging process of RBCs, the GSH–hematin adducts can be conditional formed metabolites both in health and disease.

## 5. Can the GSH S-Conjugates Be Considered Novel Metabolic Signatures in Disease States in the Upcoming Field of Metabolomics?

Precision medicine moves toward to adopt new therapeutic approaches based on the unique, individual, genetic and phenotypic response in clinical practice [241]. The human metabolome database [242] contains nearly 221,000 entries of metabolites, which are categorized by disease, age, gender, and source (serum, urine, feces, cerebrospinal fluid, sweat) and enriched with structural data. Nowadays, such a level of knowledge has been gathered in the field of metabolomics that these data should be exploited for potential biomarkers [243]. Screening for errors in metabolism is a characteristic example of the targeted metabolomic analysis [244]. Analogous tests for the disease state for adults have not been yet introduced in clinical settings [245]. A test for the early diagnosis of Parkinson’s disease, based on a dry sample of blood to be sent for MS analysis, will probably be the first such test to be introduced [246].

**Figure 3:**
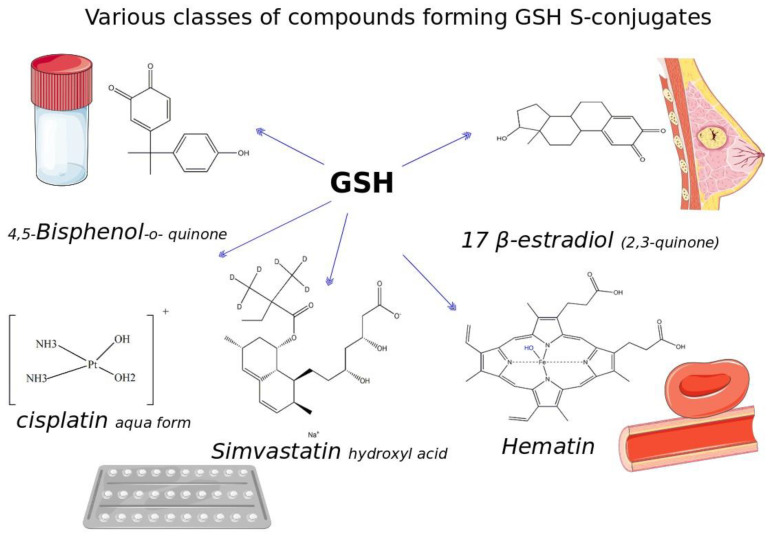
Representative compounds, of various origin, forming GSH S-conjugates are presented. In the figure, indicative pictures of the origin (source) as well as the structure of the reactant are presented to point out the diversified roles of GSH, reacting with several compounds. These include the pollutant, bisphenol A found in plasticizers and correlated with tumorigenesis, the drugs (cisplatin and simvastatin), whose conjugation with GSH is involved in chemoresistance (with diglutathionyl-platinum adducts) and elimination/clearance of the body, respectively. Furthermore, the list includes the derivatives (quinones) of 17 β-estradiol that act as promoters of tumorigenesis. The intratumor formation of catechol estrogen quinone–GSH conjugates is proposed to have a prognostic value. GSH–hematin conjugates have been detected in RBC hemolysates and may have an important role in the detoxification of free heme. Chemical structures were retrieved by PubChem, and parts of the figure were acquired by the Smart Servier Medical Art. Pubchem CIDs: Hematin (449355), Simvastatin hydroxyl acid D6 Sodium Salt (119090975), Platinum II aqua form (138393911, adjusted by ChemDraw Ultra 8.0, according to [249]), 4,5-Bisphenol-o-quinone (656690), and 17b estradiol 2–3 quinone (131769816).

GSH S-conjugates can be considered as tertiary metabolites as opposed to the essential primary metabolites and the secondary ones, which are biosynthesized upon need. As becomes evident above, GSH S-conjugates are related to the: (a) diseases’ state, (b) exposure of the body to aerial, nutritional and other chemicals (representing the exposome of the body) and (c) drugs, constituting a major part of pharmacometabolomics (Figure 3). The metabolomic analysis offers the opportunity to investigate above genomics, transcriptomics and proteomics to uncover interindividual genetic variations, not easily assessed by the other -omic approaches. The blood’s GSH levels are characterized with 20% interindividual variation, while these fluctuations persist throughout their life [27]. Moreover, the total GSH levels of the organism decrease by age [28] and by disease, as diabetes mellitus [247] and HIV infection [248]. As the formation of GSH S-conjugates is an event following serial biochemical reactions in the body, the intensity of their formation can reflect the body’s state better, discriminating (for example) responders and non-responders to a therapeutic approach, etc. Data-dependent acquisition can be achieved based on the MS signatures patterns of GSH S-conjugates. These analyses are mainly non-invasive, but the selection of the ideal GSH S-conjugates as biomarkers should take into account their clear-cut correlation with the relevant disease, stability, half-life and method(s) for samples’ preparation. 

To begin with the GSH–hematin adducts, their intensity of formation inside RBCs could be correlated with possible diagnostic and/or therapeutic interventions. Interindividual variations on GSH levels can impact the pathology of hemoglobinopathic patients or those suffering hemolytic events. Moreover, there is the case of storage-induced lesions of RBCs, related to microparticles’ formation, increased extracellular free heme concentrations, hemolytic events and NO consumption during the transfusion [250]. GSH–hematin adducts could become valuable biomarkers of the time-dependent, storage-induced aging of RBCs. 

Below, we will shortly refer to the heterogeneous studies suggesting various GSH S-conjugates and/or mercapturic acids as potential biomarkers. In the oncology field, a quinone derivative of L-DOPA reacts directly with cysteine, forming the 5-S-Cysteinyl-DOPA [251]. Many clinical studies are dedicated to assessing the prognostic value of 5-S-Cysteinyl-DOPA in melanoma patients. A 50-fold increase in its levels has been recorded at advanced stages of the disease, and 5-S-Cysteinyl-DOPA is proposed as a reliable biomarker for monitoring metastatic melanoma [252,253,254]. Moreover, the upregulated metabolism of GSH in tumors leads to the proposal of novel anticancer strategies [255]. GSH conjugation reactions with various chemotherapeutics contributing to the underlying chemoresistance should be taken into consideration in ongoing studies [113].

Cysteinyl-leukotrienes actively participate in the pathogenesis of respiratory diseases, and various strategies are already targeting them. Exhaled breath concentrate was acquired by 88 participants, who were divided into those suffering from asthma, smokers and non-smokers, as well as healthy individuals. LTD_4_ was found to be formed in significantly higher quantities in both smokers and asthmatic patients, implying that strategies decreasing its biosynthesis will potentially have therapeutic merit [256]. Moreover, the LTE_4_ levels in the urine of asthmatic patients are a better indicator of lung function than the levels of serum eosinophil cationic proteins (ECPs) [257]. There is also the case of hydroxyalkyl mercapturic acids (HAMAs), which derive from GSH conjugation to several classes of carcinogens such as ethylene oxide and acrolein. Six different HAMAs were detected in the urine of smokers; half of them were excreted in significantly higher quantities in smokers than in non-smokers [258]. On the other hand, diabetic kidneys, due to the accumulated damage, are incapable of metabolizing and excreting the diversified classes of GSH S-conjugates and their mercapturic acids, leading to their serum accumulation [259]. In addition, LTE_4_ urine levels are correlated with the pathogenesis of myocardial dysfunction [260]. Many more studies on the correlation of the GSH S-conjugates with distinct classes of pathologies are ongoing.

## 6. Conclusions

GSH, widely known for its antioxidant and redox-balancing properties, has multiple biochemical reactions involved in its metabolism. GSH S-conjugates are formed in various states of the organism both in health and disease. This list of metabolites will continue to increase, since GSH seems to offer the immediate evolutionary advantage to “recognize” and adduct to even chemical compounds, “unseen” by nature, thus far. Based on the fact that GSH-S conjugates as well as their derivatives (mercapturic acids) are the end products of a serial of mechanisms inside the body, the detection of these metabolites could be an alternative, attractive way to reflect/mirror the interindividual, genetic variabilities. Moreover, the recently characterized GSH–hematin adducts released from hemolyzed RBCs are a rich area for future research.

## Abbreviations

APAP: acetaminophen, gamma-GT: gamma-glutamyltranspeptidase, GCLC: glutamyl-cysteine ligasecatalytic subunit GPx: GSH peroxidases, GSH: glutathione, GSSG: oxidized GSH, GST: glutathione S-transferase, HIC: hemin-induced cytotoxicity, Keap1: Kelch-like ECH associated protein 1, MRP: multidrug-associated resistance protein, MS: mass spectrometry NAC: N-acetyl cysteine, NAPQI: N-acetyl p-benzoquinone imine, Nrf2: nuclear factor erythroid 2-related factor-2, PGAs: prostaglandins, RBCs: red blood cells, RNS: reactive nitrogen species, ROS: reactive oxygen species, TF: transcription factor.
